# 3′′-(2-Fluoro­benzyl­idene)-4′-(2-fluoro­phen­yl)-1′-methyl­dispiro­[acenaphthyl­ene-1,2′-pyrrolidine-3′,1′′-cyclo­penta­ne]-2,2′′-dione

**DOI:** 10.1107/S1600536812051550

**Published:** 2013-01-19

**Authors:** Gao-Zhi Chen, Xiao-Yan Wei, Yi Wang, Lu-Qing Ren, Xiao-Kun Li

**Affiliations:** a Wenzhou Medical College, School of Pharmacy, Wenzhou 325035, People’s Republic of China

## Abstract

In the title compound, C_33_H_25_F_2_NO_2_, the acenaphthene ring system forms dihedral angles of 50.93 (14) and 36.89 (14)° with the benzene rings. The pyrrolidine and cyclo­penta­none rings adopt envelope (with the N atom as the flap) and twisted conformations, respectively. In the crystal, C—H⋯O and C—H⋯F inter­actions link the mol­ecules.

## Related literature

For related structures, see: Abdul Ajees *et al.* (2002 ▶); Usha *et al.* (2003 ▶). For background to the biological properties of spiro-pyrrolidine derivatives, see: Chande *et al.* (2005 ▶); Dandia *et al.* (2003 ▶); Cravotto *et al.* (2001 ▶); Winfred *et al.* (2000 ▶); Metwally *et al.* (1998 ▶); Suenaga *et al.* (2001 ▶). For the synthesis of the title compound, see: Kumar *et al.* (2008*a* ▶,*b* ▶); Liang *et al.* (2009 ▶).
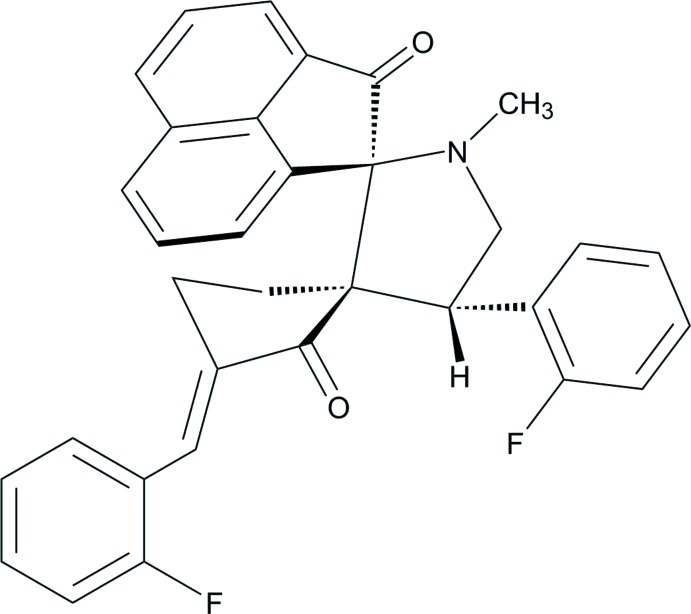


## Experimental

### 

#### Crystal data

C_33_H_25_F_2_NO_2_
*M* *_r_* = 505.54
Orthorhombic, 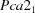

*a* = 17.728 (13) Å
*b* = 12.272 (9) Å
*c* = 12.094 (8) Å
*V* = 2631 (3) Å^3^
*Z* = 4
Mo *K*α radiation
μ = 0.09 mm^−1^
*T* = 293 K
0.45 × 0.38 × 0.27 mm

#### Data collection

Bruker SMART CCD area-detector diffractometer
Absorption correction: multi-scan (*SADABS*; Bruker, 2002 ▶) *T*_min_ = 0.641, *T*_max_ = 1.000
12188 measured reflections
2500 independent reflections
2072 reflections with *I* > 2σ(*I*)
*R*_int_ = 0.123

#### Refinement

*R*[*F* ^2^ > 2σ(*F* ^2^)] = 0.055
*wR*(*F* ^2^) = 0.126
*S* = 1.02
2500 reflections
344 parameters
13 restraints
H-atom parameters constrained
Δρ_max_ = 0.24 e Å^−3^
Δρ_min_ = −0.32 e Å^−3^

### 

Data collection: *SMART* (Bruker, 2002 ▶); cell refinement: *SAINT* (Bruker, 2002 ▶); data reduction: *SAINT*; program(s) used to solve structure: *SHELXS97* (Sheldrick, 2008 ▶); program(s) used to refine structure: *SHELXL97* (Sheldrick, 2008 ▶); molecular graphics: *SHELXTL* (Sheldrick, 2008 ▶); software used to prepare material for publication: *SHELXTL*.

## Supplementary Material

Crystal structure: contains datablock(s) I, global. DOI: 10.1107/S1600536812051550/pk2458sup1.cif

Structure factors: contains datablock(s) cd20184. DOI: 10.1107/S1600536812051550/pk2458Isup2.hkl

Additional supplementary materials:  crystallographic information; 3D view; checkCIF report

## Figures and Tables

**Table 1:** Hydrogen-bond geometry (Å, °)

*D*—H⋯*A*	*D*—H	H⋯*A*	*D*⋯*A*	*D*—H⋯*A*
C3—H3*B*⋯O1^i^	0.97	2.35	3.317 (5)	172
C14—H14*A*⋯F2^ii^	0.93	2.43	3.212 (7)	141
C25—H25⋯O2^iii^	0.93	2.58	3.458 (7)	158

Symmetry codes: (i) 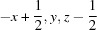; (ii) 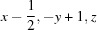; (iii) 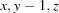.

**Fig. 1.**
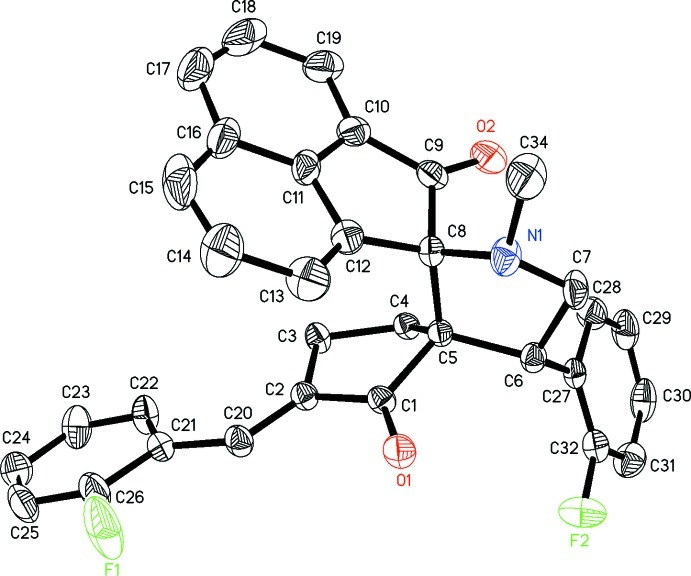
The molecular structure of the title compound, showing 30% displacement ellipsoids for the non-hydrogen atoms. Hydrogen atoms are drawn as spheres of arbitrary radius.

## supplementary crystallographic information

### Comment

Spiro-pyrrolidine compounds find applications in the synthesis of biologically active compounds. The synthesis of spiro compounds has drawn considerable attention of chemists, in view of their wide spectrum of pharmacological properties (Chande *et al.*, 2005; Dandia *et al.*, 2003; Cravotto *et al.*, 2001; Winfred *et al.*, 2000; Metwally *et al.*, 1998; Suenaga *et al.*, 2001). In the present study, the 1,3-dipolar cycloaddition of an azomethine ylide generated *in situ* from acenaphthenequinone and sarcosine to novel mono-carbonyl analogue of curcumin containg cyclopentanone afforded the title compound (Kumar *et al.*, 2008; Liang *et al.*, 2009). With this background, and in continuation of our structural analysis of spiro-pyrrolidine derivatives, the X-ray crystal structure determination of the title compound, (I), was undertaken.

The bond lengths and angles in the pyrrolidine ring are slightly larger than normal values because of bulky substituents on the pyrrolidine moiety. A similar effect has been observed in related reported structures (Abdul Ajees *et al.*, 2002; Usha *et al.*, 2003). The sum of the angles at atom N1 [339.1 (11)°] is in accordance with *sp*^3^-hybridization. The dihedral angles between the acenaphthene ring system and phenyl rings C21—C26 and C27—C32 are 50.93 (14)° and 36.89 (14)° respectively, while that between the two phenyl-ring substituents is 87.55 (17)°. The pyrrolidine and cyclopentanone ring both adopt an envelope conformation. In addition to van der Waals interactions, the crystal structure is stabilized by C—H···O and C—H···F intramolecular interactions. In the present study, the 1,3-dipolar cycloaddition of an azomethine ylide generated *in situ* from acenaphthenequinone and sarcosine to novel mono-carbonyl analogue of curcumin containg cyclopentanone afforded title compound.

### Experimental

A mixture of (2E,5E)-2,5-bis(2-fluorobenzylidene)cyclopentanone (1 mmol), (Liang *et al.*, 2009), acenaphthenequinone (0.182 g, 1 mmol), and sarcosine (0.089 g, 1 mmol) was dissolved in methanol (10 mL) and refluxed for 1 h. After completion of the reaction as evident from TLC, the mixture was cooled to room temperature and poured into cold water (50 mL). The precipitate was filtered and washed with water to obtain pure product as a yellow solid (79.6% yield, mp 93.2–95.0°C). Single crystals were grown in an ethyl acetate/CH_2_Cl_2_ mixture (2:1ν/ν).

### Refinement

The H(C) atom positions were calculated. The H atoms bound to C were positioned geometrically and allowed to ride on their parent atoms at distances of 0.96 Å (RCH_3_), 0.97 Å (R_2_CH_2_), 0.98 Å (R_3_CH), 0.93 Å (R_2_CH), and with U_iso_(H) values set to either 1.2U_eq_ or 1.5U_eq_ (RCH_3_) of the attached atom.

### Crystal data

**Table:**

C_33_H_25_F_2_NO_2_	*F*(000) = 1052
*M**_r_* = 505.54	*D*_x_ = 1.274 Mg m^−^^3^
Orthorhombic, *P**c**a*2_1_	Mo *K*α radiation, λ = 0.71073 Å
Hall symbol: P 2c -2ac	Cell parameters from 3224 reflections
*a* = 17.728 (13) Å	θ = 4.6–41.9°
*b* = 12.272 (9) Å	µ = 0.09 mm^−^^1^
*c* = 12.094 (8) Å	*T* = 293 K
*V* = 2631 (3) Å^3^	Prismatic, yellow
*Z* = 4	0.45 × 0.38 × 0.27 mm

### Data collection

**Table:**

Bruker SMART CCD area-detector diffractometer	2500 independent reflections
Radiation source: fine-focus sealed tube	2072 reflections with *I* > 2σ(*I*)
Graphite monochromator	*R*_int_ = 0.123
φ and ω scans	θ_max_ = 25.5°, θ_min_ = 1.7°
Absorption correction: multi-scan (*SADABS*; Bruker, 2002)	*h* = −21→20
*T*_min_ = 0.641, *T*_max_ = 1.000	*k* = −14→10
12188 measured reflections	*l* = −14→13

### Refinement

**Table:**

Refinement on *F*^2^	Primary atom site location: structure-invariant direct methods
Least-squares matrix: full	Secondary atom site location: difference Fourier map
*R*[*F*^2^ > 2σ(*F*^2^)] = 0.055	Hydrogen site location: inferred from neighbouring sites
*wR*(*F*^2^) = 0.126	H-atom parameters constrained
*S* = 1.02	*w* = 1/[σ^2^(*F*_o_^2^) + (0.0837*P*)^2^] where *P* = (*F*_o_^2^ + 2*F*_c_^2^)/3
2500 reflections	(Δ/σ)_max_ < 0.001
344 parameters	Δρ_max_ = 0.24 e Å^−^^3^
13 restraints	Δρ_min_ = −0.32 e Å^−^^3^

### Special details

**Table:**

Geometry. All esds (except the esd in the dihedral angle between two l.s. planes) are estimated using the full covariance matrix. The cell esds are taken into account individually in the estimation of esds in distances, angles and torsion angles; correlations between esds in cell parameters are only used when they are defined by crystal symmetry. An approximate (isotropic) treatment of cell esds is used for estimating esds involving l.s. planes.
Refinement. Refinement of *F*^2^ against ALL reflections. The weighted *R*-factor *wR* and goodness of fit *S* are based on *F*^2^, conventional *R*-factors *R* are based on *F*, with *F* set to zero for negative *F*^2^. The threshold expression of *F*^2^ > σ(*F*^2^) is used only for calculating *R*-factors(gt) *etc*. and is not relevant to the choice of reflections for refinement. *R*-factors based on *F*^2^ are statistically about twice as large as those based on *F*, and *R*- factors based on ALL data will be even larger.

### Fractional atomic coordinates and isotropic or equivalent isotropic displacement parameters (Å^2^)

**Table:**

	*x*	*y*	*z*	*U*_iso_*/*U*_eq_	
F1	0.1562 (3)	0.1946 (2)	0.9893 (3)	0.1212 (15)	
F2	0.37848 (15)	0.7382 (2)	1.1356 (2)	0.0723 (8)	
O1	0.20514 (17)	0.54755 (19)	1.1294 (2)	0.0503 (7)	
O2	0.08624 (18)	0.9035 (2)	0.9244 (3)	0.0656 (9)	
N1	0.0970 (2)	0.7803 (3)	1.1430 (3)	0.0494 (8)	
C1	0.1917 (2)	0.5827 (3)	1.0368 (3)	0.0351 (8)	
C2	0.1848 (2)	0.5181 (3)	0.9340 (3)	0.0367 (8)	
C3	0.1787 (2)	0.5964 (3)	0.8387 (3)	0.0390 (8)	
H3A	0.1272	0.6007	0.8120	0.047*	
H3B	0.2113	0.5745	0.7783	0.047*	
C4	0.2041 (2)	0.7055 (2)	0.8874 (3)	0.0351 (8)	
H4A	0.1802	0.7655	0.8486	0.042*	
H4B	0.2583	0.7135	0.8812	0.042*	
C5	0.18001 (19)	0.7045 (2)	1.0097 (3)	0.0329 (7)	
C6	0.2244 (2)	0.7800 (3)	1.0916 (3)	0.0376 (8)	
H6	0.2461	0.7324	1.1483	0.045*	
C7	0.1640 (2)	0.8486 (3)	1.1487 (3)	0.0515 (10)	
H7A	0.1777	0.8636	1.2249	0.062*	
H7B	0.1562	0.9170	1.1103	0.062*	
C8	0.0945 (2)	0.7332 (3)	1.0318 (3)	0.0376 (8)	
C9	0.0633 (2)	0.8121 (3)	0.9403 (3)	0.0460 (9)	
C10	0.0007 (2)	0.7571 (4)	0.8828 (3)	0.0520 (11)	
C11	−0.0125 (2)	0.6583 (3)	0.9373 (3)	0.0501 (10)	
C12	0.0375 (2)	0.6408 (3)	1.0259 (3)	0.0467 (9)	
C13	0.0260 (3)	0.5518 (4)	1.0922 (4)	0.0690 (13)	
H14	0.0563	0.5400	1.1538	0.083*	
C14	−0.0322 (4)	0.4789 (5)	1.0659 (6)	0.0884 (17)	
H14A	−0.0390	0.4182	1.1109	0.106*	
C15	−0.0788 (3)	0.4923 (4)	0.9792 (6)	0.093 (2)	
H15	−0.1158	0.4407	0.9646	0.112*	
C16	−0.0713 (3)	0.5851 (4)	0.9099 (4)	0.0718 (14)	
C17	−0.1165 (3)	0.6177 (6)	0.8195 (6)	0.094 (2)	
H17	−0.1559	0.5731	0.7967	0.113*	
C18	−0.1034 (3)	0.7133 (7)	0.7648 (5)	0.098 (2)	
H18	−0.1339	0.7309	0.7048	0.118*	
C19	−0.0455 (3)	0.7867 (4)	0.7954 (4)	0.0719 (15)	
H19	−0.0387	0.8523	0.7582	0.086*	
C20	0.1819 (2)	0.4093 (3)	0.9397 (3)	0.0430 (8)	
H21	0.1849	0.3811	1.0109	0.052*	
C21	0.1747 (2)	0.3280 (3)	0.8532 (3)	0.0452 (9)	
C22	0.1803 (3)	0.3482 (3)	0.7384 (3)	0.0562 (11)	
H22	0.1859	0.4196	0.7140	0.067*	
C23	0.1776 (3)	0.2659 (4)	0.6621 (4)	0.0689 (14)	
H23	0.1820	0.2824	0.5873	0.083*	
C24	0.1685 (3)	0.1603 (4)	0.6939 (5)	0.0726 (14)	
H24	0.1678	0.1048	0.6415	0.087*	
C25	0.1604 (4)	0.1365 (4)	0.8058 (5)	0.0829 (18)	
H25	0.1528	0.0652	0.8294	0.099*	
C26	0.1639 (3)	0.2197 (3)	0.8797 (4)	0.0676 (14)	
C27	0.2887 (2)	0.8456 (3)	1.0442 (3)	0.0374 (8)	
C28	0.2768 (3)	0.9352 (3)	0.9739 (3)	0.0533 (11)	
H28	0.2278	0.9556	0.9559	0.064*	
C29	0.3368 (3)	0.9934 (3)	0.9313 (4)	0.0632 (12)	
H29	0.3274	1.0522	0.8849	0.076*	
C30	0.4090 (3)	0.9666 (4)	0.9558 (4)	0.0647 (13)	
H30	0.4485	1.0070	0.9263	0.078*	
C31	0.4239 (3)	0.8797 (4)	1.0239 (4)	0.0614 (11)	
H31	0.4731	0.8598	1.0412	0.074*	
C32	0.3623 (2)	0.8225 (3)	1.0661 (3)	0.0457 (9)	
C33	0.0275 (3)	0.8304 (4)	1.1823 (4)	0.0793 (15)	
H33A	0.0325	0.8479	1.2593	0.119*	
H33B	−0.0136	0.7805	1.1724	0.119*	
H33C	0.0179	0.8958	1.1410	0.119*	

### Atomic displacement parameters (Å^2^)

**Table:**

	*U*^11^	*U*^22^	*U*^33^	*U*^12^	*U*^13^	*U*^23^
F1	0.251 (5)	0.0477 (15)	0.0652 (18)	−0.031 (2)	−0.035 (2)	0.0173 (15)
F2	0.0645 (19)	0.0727 (17)	0.0798 (18)	0.0172 (13)	0.0031 (14)	0.0249 (15)
O1	0.078 (2)	0.0374 (14)	0.0354 (13)	−0.0033 (12)	−0.0117 (13)	0.0106 (11)
O2	0.073 (2)	0.0410 (16)	0.082 (2)	0.0067 (14)	−0.0102 (17)	0.0123 (16)
N1	0.050 (2)	0.060 (2)	0.0389 (16)	−0.0045 (15)	0.0072 (14)	−0.0143 (15)
C1	0.041 (2)	0.0303 (17)	0.0343 (18)	−0.0005 (14)	0.0016 (15)	0.0056 (15)
C2	0.043 (2)	0.0327 (18)	0.0347 (17)	−0.0015 (14)	0.0011 (15)	0.0035 (15)
C3	0.057 (3)	0.0307 (18)	0.0296 (16)	−0.0015 (15)	0.0021 (16)	0.0000 (14)
C4	0.045 (2)	0.0305 (17)	0.0303 (16)	0.0003 (14)	0.0002 (14)	0.0042 (14)
C5	0.039 (2)	0.0298 (17)	0.0298 (16)	0.0002 (14)	−0.0024 (14)	−0.0003 (14)
C6	0.046 (2)	0.0338 (17)	0.0326 (16)	−0.0041 (14)	−0.0044 (16)	−0.0004 (14)
C7	0.058 (3)	0.054 (2)	0.043 (2)	−0.0043 (18)	0.0014 (18)	−0.0198 (19)
C8	0.041 (2)	0.0350 (18)	0.0366 (17)	−0.0028 (14)	−0.0019 (16)	−0.0044 (15)
C9	0.052 (3)	0.039 (2)	0.046 (2)	0.0113 (17)	0.0017 (19)	−0.0034 (17)
C10	0.049 (3)	0.063 (3)	0.045 (2)	0.0167 (19)	−0.0042 (19)	−0.0184 (19)
C11	0.044 (2)	0.054 (2)	0.053 (2)	−0.0005 (16)	0.0025 (19)	−0.024 (2)
C12	0.043 (2)	0.045 (2)	0.052 (2)	−0.0027 (16)	0.0068 (19)	−0.0049 (18)
C13	0.057 (3)	0.068 (3)	0.082 (3)	−0.012 (2)	0.017 (2)	0.017 (3)
C14	0.075 (4)	0.076 (4)	0.114 (5)	−0.032 (3)	0.013 (4)	0.007 (3)
C15	0.078 (4)	0.069 (3)	0.133 (5)	−0.038 (3)	0.027 (4)	−0.028 (4)
C16	0.047 (3)	0.086 (4)	0.082 (3)	−0.005 (2)	0.001 (2)	−0.043 (3)
C17	0.067 (4)	0.115 (5)	0.101 (5)	−0.001 (3)	−0.019 (3)	−0.060 (4)
C18	0.077 (4)	0.149 (6)	0.069 (4)	0.031 (4)	−0.037 (3)	−0.055 (4)
C19	0.071 (4)	0.094 (4)	0.050 (2)	0.031 (3)	−0.014 (2)	−0.018 (2)
C20	0.055 (2)	0.0339 (19)	0.0407 (18)	−0.0037 (15)	−0.0023 (17)	0.0052 (16)
C21	0.048 (2)	0.035 (2)	0.053 (2)	−0.0003 (16)	−0.0060 (18)	−0.0006 (17)
C22	0.073 (3)	0.043 (2)	0.052 (2)	−0.016 (2)	0.004 (2)	−0.0028 (19)
C23	0.080 (4)	0.068 (3)	0.058 (3)	−0.020 (2)	0.009 (2)	−0.018 (2)
C24	0.081 (4)	0.057 (3)	0.080 (4)	0.011 (2)	−0.018 (3)	−0.032 (3)
C25	0.131 (5)	0.032 (2)	0.085 (4)	0.004 (2)	−0.040 (3)	−0.004 (3)
C26	0.109 (4)	0.035 (2)	0.059 (3)	−0.004 (2)	−0.024 (3)	0.008 (2)
C27	0.048 (2)	0.0332 (18)	0.0313 (16)	−0.0048 (14)	−0.0008 (15)	−0.0074 (15)
C28	0.067 (3)	0.040 (2)	0.052 (2)	−0.0045 (18)	−0.006 (2)	0.0060 (18)
C29	0.090 (4)	0.038 (2)	0.061 (3)	−0.022 (2)	−0.003 (3)	0.002 (2)
C30	0.083 (4)	0.061 (3)	0.050 (3)	−0.037 (2)	0.009 (2)	−0.011 (2)
C31	0.050 (3)	0.073 (3)	0.061 (3)	−0.005 (2)	0.004 (2)	−0.009 (2)
C32	0.053 (3)	0.040 (2)	0.044 (2)	−0.0025 (17)	0.0018 (18)	−0.0059 (17)
C33	0.058 (3)	0.106 (4)	0.074 (3)	0.000 (3)	0.014 (3)	−0.039 (3)

### Geometric parameters (Å, º)

**Table:**

F1—C26	1.368 (6)	C14—H14A	0.9300
F2—C32	1.362 (5)	C15—C16	1.421 (8)
O1—C1	1.224 (4)	C15—H15	0.9300
O2—C9	1.209 (5)	C16—C17	1.413 (9)
N1—C7	1.455 (5)	C17—C18	1.367 (9)
N1—C33	1.456 (6)	C17—H17	0.9300
N1—C8	1.465 (5)	C18—C19	1.415 (8)
C1—C2	1.478 (5)	C18—H18	0.9300
C1—C5	1.545 (5)	C19—H19	0.9300
C2—C20	1.338 (5)	C20—C21	1.451 (5)
C2—C3	1.505 (5)	C20—H21	0.9300
C3—C4	1.530 (5)	C21—C26	1.380 (6)
C3—H3A	0.9700	C21—C22	1.414 (6)
C3—H3B	0.9700	C22—C23	1.369 (6)
C4—C5	1.539 (5)	C22—H22	0.9300
C4—H4A	0.9700	C23—C24	1.361 (7)
C4—H4B	0.9700	C23—H23	0.9300
C5—C6	1.568 (5)	C24—C25	1.392 (8)
C5—C8	1.580 (5)	C24—H24	0.9300
C6—C27	1.508 (5)	C25—C26	1.359 (6)
C6—C7	1.527 (5)	C25—H25	0.9300
C6—H6	0.9800	C27—C32	1.362 (5)
C7—H7A	0.9700	C27—C28	1.405 (5)
C7—H7B	0.9700	C28—C29	1.381 (6)
C8—C12	1.520 (5)	C28—H28	0.9300
C8—C9	1.571 (5)	C29—C30	1.353 (7)
C9—C10	1.473 (6)	C29—H29	0.9300
C10—C19	1.386 (6)	C30—C31	1.373 (7)
C10—C11	1.400 (6)	C30—H30	0.9300
C11—C12	1.408 (6)	C31—C32	1.395 (6)
C11—C16	1.415 (6)	C31—H31	0.9300
C12—C13	1.370 (6)	C33—H33A	0.9600
C13—C14	1.402 (7)	C33—H33B	0.9600
C13—H14	0.9300	C33—H33C	0.9600
C14—C15	1.344 (10)		
			
C7—N1—C33	115.6 (3)	C13—C14—H14A	118.3
C7—N1—C8	107.2 (3)	C14—C15—C16	120.1 (5)
C33—N1—C8	116.1 (4)	C14—C15—H15	120.0
O1—C1—C2	126.6 (3)	C16—C15—H15	120.0
O1—C1—C5	124.1 (3)	C17—C16—C11	114.8 (6)
C2—C1—C5	109.3 (3)	C17—C16—C15	129.1 (5)
C20—C2—C1	119.7 (3)	C11—C16—C15	116.0 (5)
C20—C2—C3	132.3 (3)	C18—C17—C16	121.4 (5)
C1—C2—C3	107.9 (3)	C18—C17—H17	119.3
C2—C3—C4	104.1 (3)	C16—C17—H17	119.3
C2—C3—H3A	110.9	C17—C18—C19	122.9 (5)
C4—C3—H3A	110.9	C17—C18—H18	118.6
C2—C3—H3B	110.9	C19—C18—H18	118.6
C4—C3—H3B	110.9	C10—C19—C18	117.5 (6)
H3A—C3—H3B	109.0	C10—C19—H19	121.3
C3—C4—C5	106.3 (3)	C18—C19—H19	121.3
C3—C4—H4A	110.5	C2—C20—C21	130.8 (3)
C5—C4—H4A	110.5	C2—C20—H21	114.6
C3—C4—H4B	110.5	C21—C20—H21	114.6
C5—C4—H4B	110.5	C26—C21—C22	113.9 (4)
H4A—C4—H4B	108.7	C26—C21—C20	120.5 (4)
C4—C5—C1	100.0 (3)	C22—C21—C20	125.5 (4)
C4—C5—C6	117.6 (3)	C23—C22—C21	122.1 (4)
C1—C5—C6	111.8 (3)	C23—C22—H22	119.0
C4—C5—C8	115.3 (3)	C21—C22—H22	119.0
C1—C5—C8	108.0 (3)	C24—C23—C22	121.0 (5)
C6—C5—C8	104.1 (3)	C24—C23—H23	119.5
C27—C6—C7	114.1 (3)	C22—C23—H23	119.5
C27—C6—C5	117.0 (3)	C23—C24—C25	119.2 (4)
C7—C6—C5	105.1 (3)	C23—C24—H24	120.4
C27—C6—H6	106.7	C25—C24—H24	120.4
C7—C6—H6	106.7	C26—C25—C24	118.4 (5)
C5—C6—H6	106.7	C26—C25—H25	120.8
N1—C7—C6	103.5 (3)	C24—C25—H25	120.8
N1—C7—H7A	111.1	C25—C26—F1	117.6 (4)
C6—C7—H7A	111.1	C25—C26—C21	125.3 (4)
N1—C7—H7B	111.1	F1—C26—C21	117.1 (4)
C6—C7—H7B	111.1	C32—C27—C28	115.1 (3)
H7A—C7—H7B	109.0	C32—C27—C6	122.6 (3)
N1—C8—C12	110.9 (3)	C28—C27—C6	122.2 (3)
N1—C8—C9	114.5 (3)	C29—C28—C27	120.9 (4)
C12—C8—C9	101.2 (3)	C29—C28—H28	119.5
N1—C8—C5	102.4 (3)	C27—C28—H28	119.5
C12—C8—C5	117.6 (3)	C30—C29—C28	121.4 (4)
C9—C8—C5	110.9 (3)	C30—C29—H29	119.3
O2—C9—C10	127.2 (4)	C28—C29—H29	119.3
O2—C9—C8	124.4 (4)	C29—C30—C31	120.1 (4)
C10—C9—C8	108.4 (3)	C29—C30—H30	119.9
C19—C10—C11	119.2 (5)	C31—C30—H30	119.9
C19—C10—C9	133.2 (5)	C30—C31—C32	117.4 (4)
C11—C10—C9	107.5 (3)	C30—C31—H31	121.3
C10—C11—C12	112.7 (3)	C32—C31—H31	121.3
C10—C11—C16	124.2 (4)	C27—C32—F2	118.7 (3)
C12—C11—C16	123.1 (4)	C27—C32—C31	125.0 (4)
C13—C12—C11	118.3 (4)	F2—C32—C31	116.3 (4)
C13—C12—C8	131.8 (4)	N1—C33—H33A	109.5
C11—C12—C8	109.9 (3)	N1—C33—H33B	109.5
C12—C13—C14	119.1 (5)	H33A—C33—H33B	109.5
C12—C13—H14	120.5	N1—C33—H33C	109.5
C14—C13—H14	120.5	H33A—C33—H33C	109.5
C15—C14—C13	123.4 (5)	H33B—C33—H33C	109.5
C15—C14—H14A	118.3		
			
O1—C1—C2—C20	10.5 (6)	C16—C11—C12—C13	−3.8 (6)
C5—C1—C2—C20	−170.2 (3)	C10—C11—C12—C8	−3.1 (5)
O1—C1—C2—C3	−172.3 (4)	C16—C11—C12—C8	179.1 (4)
C5—C1—C2—C3	7.0 (4)	N1—C8—C12—C13	−49.0 (5)
C20—C2—C3—C4	−168.1 (4)	C9—C8—C12—C13	−170.8 (4)
C1—C2—C3—C4	15.2 (4)	C5—C8—C12—C13	68.3 (6)
C2—C3—C4—C5	−32.0 (4)	N1—C8—C12—C11	127.7 (3)
C3—C4—C5—C1	34.8 (4)	C9—C8—C12—C11	5.8 (4)
C3—C4—C5—C6	156.0 (3)	C5—C8—C12—C11	−115.1 (3)
C3—C4—C5—C8	−80.6 (3)	C11—C12—C13—C14	3.4 (6)
O1—C1—C5—C4	153.6 (4)	C8—C12—C13—C14	179.9 (5)
C2—C1—C5—C4	−25.7 (3)	C12—C13—C14—C15	−1.0 (9)
O1—C1—C5—C6	28.4 (5)	C13—C14—C15—C16	−1.3 (10)
C2—C1—C5—C6	−150.9 (3)	C10—C11—C16—C17	1.4 (6)
O1—C1—C5—C8	−85.5 (4)	C12—C11—C16—C17	178.9 (4)
C2—C1—C5—C8	95.2 (3)	C10—C11—C16—C15	−176.1 (4)
C4—C5—C6—C27	−1.6 (4)	C12—C11—C16—C15	1.5 (6)
C1—C5—C6—C27	113.3 (3)	C14—C15—C16—C17	−175.9 (6)
C8—C5—C6—C27	−130.4 (3)	C14—C15—C16—C11	1.0 (8)
C4—C5—C6—C7	126.1 (3)	C11—C16—C17—C18	−0.4 (8)
C1—C5—C6—C7	−119.0 (3)	C15—C16—C17—C18	176.6 (6)
C8—C5—C6—C7	−2.7 (3)	C16—C17—C18—C19	−1.2 (9)
C33—N1—C7—C6	−174.1 (4)	C11—C10—C19—C18	−1.0 (6)
C8—N1—C7—C6	−42.8 (4)	C9—C10—C19—C18	−177.1 (4)
C27—C6—C7—N1	155.9 (3)	C17—C18—C19—C10	1.9 (8)
C5—C6—C7—N1	26.4 (4)	C1—C2—C20—C21	179.5 (4)
C7—N1—C8—C12	166.6 (3)	C3—C2—C20—C21	3.0 (7)
C33—N1—C8—C12	−62.4 (5)	C2—C20—C21—C26	−172.3 (4)
C7—N1—C8—C9	−79.7 (4)	C2—C20—C21—C22	9.8 (7)
C33—N1—C8—C9	51.3 (5)	C26—C21—C22—C23	−2.1 (7)
C7—N1—C8—C5	40.4 (4)	C20—C21—C22—C23	175.8 (4)
C33—N1—C8—C5	171.4 (4)	C21—C22—C23—C24	0.7 (7)
C4—C5—C8—N1	−151.9 (3)	C22—C23—C24—C25	1.3 (8)
C1—C5—C8—N1	97.2 (3)	C23—C24—C25—C26	−1.7 (9)
C6—C5—C8—N1	−21.7 (3)	C24—C25—C26—F1	−179.6 (6)
C4—C5—C8—C12	86.3 (4)	C24—C25—C26—C21	0.2 (9)
C1—C5—C8—C12	−24.6 (4)	C22—C21—C26—C25	1.7 (7)
C6—C5—C8—C12	−143.5 (3)	C20—C21—C26—C25	−176.4 (5)
C4—C5—C8—C9	−29.4 (4)	C22—C21—C26—F1	−178.5 (5)
C1—C5—C8—C9	−140.2 (3)	C20—C21—C26—F1	3.4 (7)
C6—C5—C8—C9	100.8 (3)	C7—C6—C27—C32	128.5 (4)
N1—C8—C9—O2	51.8 (5)	C5—C6—C27—C32	−108.3 (4)
C12—C8—C9—O2	171.1 (4)	C7—C6—C27—C28	−51.5 (4)
C5—C8—C9—O2	−63.4 (4)	C5—C6—C27—C28	71.7 (4)
N1—C8—C9—C10	−125.9 (3)	C32—C27—C28—C29	0.3 (5)
C12—C8—C9—C10	−6.6 (4)	C6—C27—C28—C29	−179.7 (4)
C5—C8—C9—C10	118.9 (3)	C27—C28—C29—C30	−0.2 (7)
O2—C9—C10—C19	4.0 (7)	C28—C29—C30—C31	0.2 (7)
C8—C9—C10—C19	−178.4 (4)	C29—C30—C31—C32	−0.4 (6)
O2—C9—C10—C11	−172.4 (4)	C28—C27—C32—F2	178.6 (3)
C8—C9—C10—C11	5.2 (4)	C6—C27—C32—F2	−1.4 (5)
C19—C10—C11—C12	−178.4 (4)	C28—C27—C32—C31	−0.6 (5)
C9—C10—C11—C12	−1.4 (5)	C6—C27—C32—C31	179.5 (3)
C19—C10—C11—C16	−0.7 (6)	C30—C31—C32—C27	0.6 (6)
C9—C10—C11—C16	176.4 (4)	C30—C31—C32—F2	−178.5 (3)
C10—C11—C12—C13	174.0 (4)		

### Hydrogen-bond geometry (Å, º)

**Table:**

*D*—H···*A*	*D*—H	H···*A*	*D*···*A*	*D*—H···*A*
C3—H3*B*···O1^i^	0.97	2.35	3.317 (5)	172
C14—H14*A*···F2^ii^	0.93	2.43	3.212 (7)	141
C25—H25···O2^iii^	0.93	2.58	3.458 (7)	158

Symmetry codes: (i) −*x*+1/2, *y*, *z*−1/2; (ii) *x*−1/2, −*y*+1, *z*; (iii) *x*, *y*−1, *z*.
